# Supplementary material for: Nutritional Quality of Fast Food Kids Meals and Their Contribution to the Diets of School-Aged Children
Source: Nutrients. 2020 Feb 26;12(3):612. doi: 10.3390/nu12030612 (PMC7146270; doi:10.3390/nu12030612)
Supplement: Supplementary file 1 [file nutrients-12-00612-s001.pdf]

Supplementary Material:

Table S1. Recommended Daily Intake for Moderate Activity (kcal)[1,2]

Meal time	Nutritional content	Preschool age		School age		Middle school age	
		Girls	Boys	Girls	Boys	Girls	Boys
Breakfast	Total energy	325 (260,390)	313 (250,375)	482 (386,579)	445 (356,534)	694 (555,833)	578 (463,694)
	Proteins	49 (39,59)	47 (38,56)	72 (58,87)	67 (53,80)	104 (83,125)	87 (70,104)
	Carbohydrates	179 (143,215)	172 (138,206)	265 (212,318)	245 (196,294)	382 (305,458)	318 (254,382)
	Lipids	98 (78,117)	94 (75,113)	145 (116,174)	133 (107,160)	208 (167,250)	173 (139,208)
Lunch	Total energy	390 (325,455)	375 (313,438)	579 (482,675)	534 (445,623)	833 (694,971)	694 (578,809)
	Proteins	59 (49,69)	56 (47,66)	87 (72,101)	80 (67,93)	125 (104,146)	104 (87,121)
	Carbohydrates	215 (179,250)	206 (172,241)	318 (265,371)	294 (245,343)	458 (382,534)	382 (318,445)
	Lipids	117 (98,137)	113 (94,131)	174 (145,203)	160 (133,187)	250 (208,291)	208 (173,243)
Snack	Total energy	195 (130,260)	188 (125,250)	289 (193,386)	267 (178,356)	416 (278,555)	347 (231,463)
	Proteins	29 (20,39)	28 (19,38)	43 (29,58)	40 (27,53)	62 (42,83)	52 (35,69)
	Carbohydrates	107 (72,143)	103 (69,138)	159 (106,212)	147 (98,196)	229 (153,305)	191 (127,254)
	Lipids	59 (39,78)	56 (38,75)	87 (58,116)	80 (53,107)	125 (83,167)	104 (69,139)

Source: Bourges et al. (2008).

(1) In parentheses interval (+/- 5%).

(2) Energy contribution considering a breakdown of 55% carbohydrates, 15% proteins, and 30% lipids according to the total daily energetic requirements by school group age and gender.
